# Supplementary material for: Cryo-tomography reveals rigid-body motion and organization of apicomplexan invasion machinery
Source: Nat Commun. 2023 Mar 30;14:1775. doi: 10.1038/s41467-023-37327-w (PMC10063558; doi:10.1038/s41467-023-37327-w)
Supplement: Supplementary file 1 — Supplementary Information [file 41467_2023_37327_MOESM1_ESM.pdf]

Supplementary Information for: Gui, et al. Cryo-tomography reveals rigid-body motion and organization of apicomplexan invasion machinery

The PDF file includes:

Supplementary Figures 1 to 8

Supplementary Tables 1 to 2

Other Supplementary Material for this manuscript includes the following:

Supplementary Movie 1

Supplementary Figures

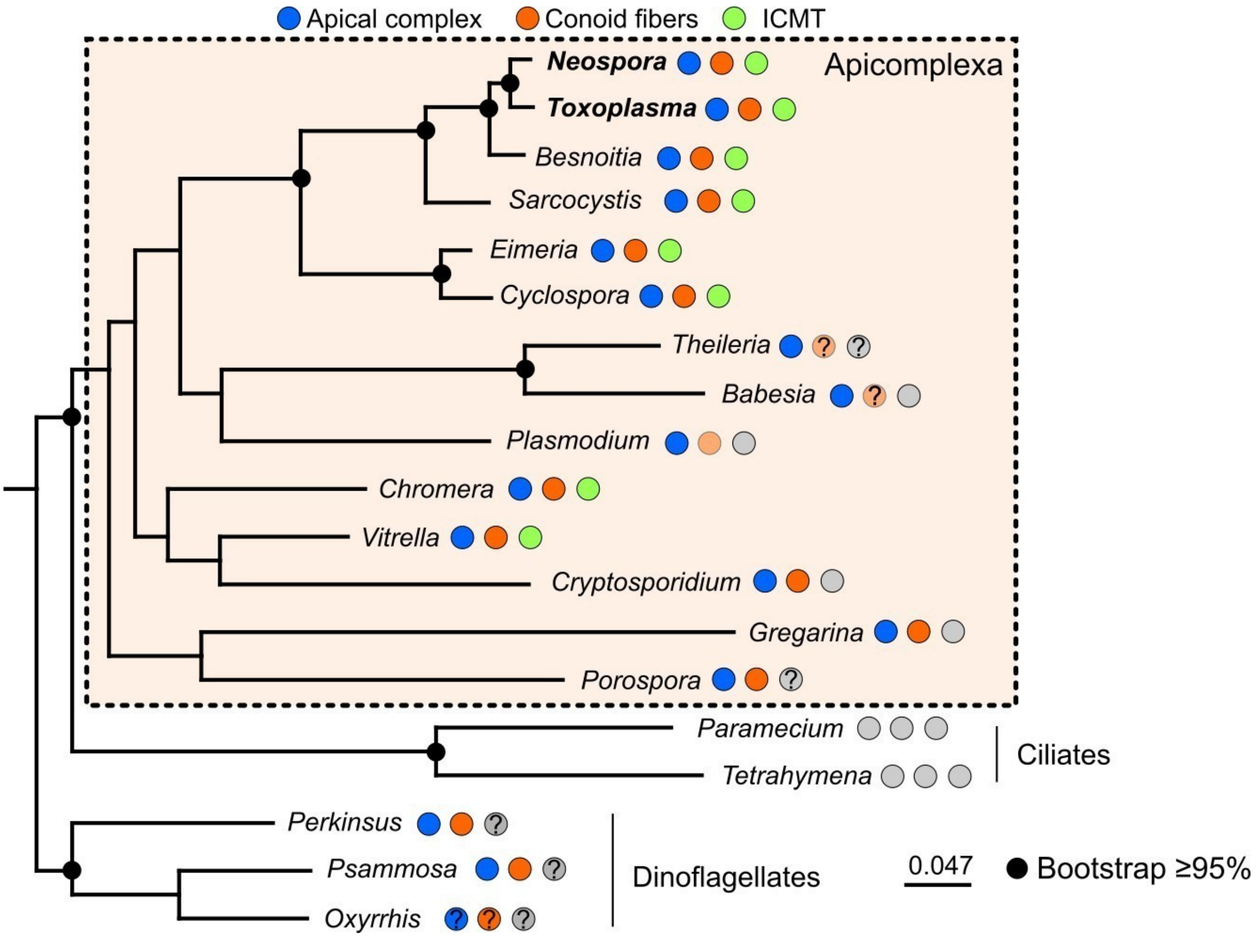

Supplementary Figure 1. Phylogenetic comparison of Alveolate organisms. Phylogenetic tree of Apicomplexa and other alveolate organisms including representative ciliates and dinoflagellates. The presence of components of the apical complex is noted with colored circles next to each organism name. Note that the conoid tubulin fibers appear to be present in some, but not all, lifecycle stages of the Aconoidasida (*e.g. Plasmodium, Babesia*) that have a conoid structure (light orange circles).

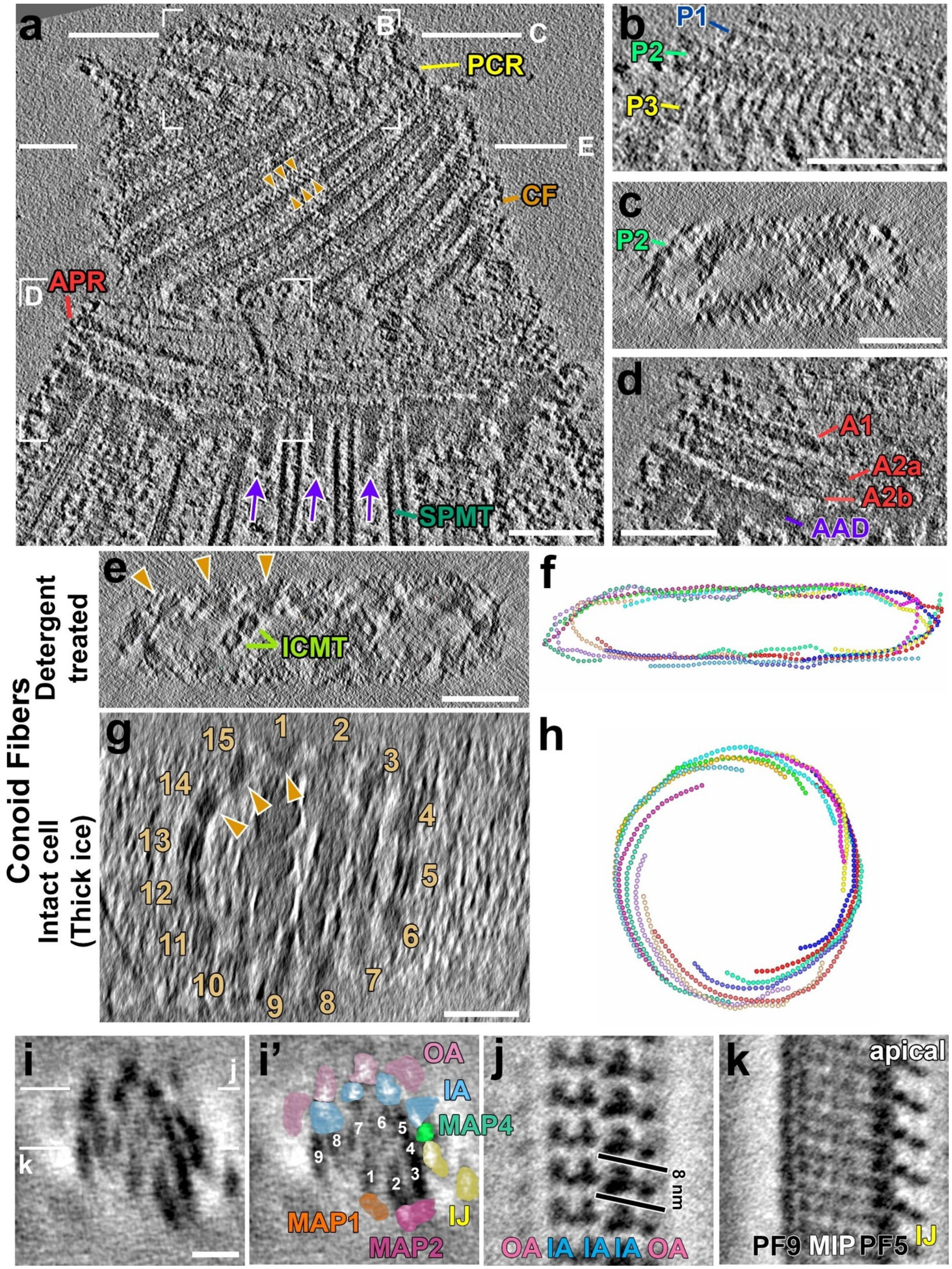

Supplementary Figure 2. Detergent-extracted *Toxoplasma* exhibits a flattened structure in tomograms. (a-b) Tomographic slices of the apical complex of detergent-extracted tachyzoites in longitudinal (a, b, d) and cross-sectional orientation (c). White lines in (a) indicate the positions of the cross-sectional views shown in (c and e). Note the periodic connections between neighboring CFs (orange arrowheads in (a)). Other labels and coloring as for Figure 1. White boxes in (a) indicate the regions magnified and orientation-optimized in (b and d). (b) shows the detailed PCR structure with three layers (P1-P3) and repeating densities connecting the layers. A

cross-sectional view of PCR-P2 (c) reveals severe flattening of the detergent- extracted sample. The APR-region is shown in (d) with at least three layers of APRs (A1, A2a, A2b) and a ring of amorphous APR-associated density (AAD). **(e-h)** Comparison of tomographic slices (e, g) and graphical models (f, h) of cross-sectional views of the apical complex in a detergent-extracted, severely flattened and distorted *Toxoplasma* cell (e, f), and in a native, un-flattened *N. caninum* cell that was embedded in a relatively thick ice layer and cryo- FIB milled to avoid cell flattening (g, h). Viewed from the apical end; dots/model points in (f, h) indicate repeat units of the CFs (orange arrowheads/1-15 in (e, g)). **(i-k)** Cross-sectional (i: original, i': pseudo-colored) and longitudinal (j, k) tomographic slices through the subtomogram averaged 8-nm repeats of the conoid fibers in detergent-extracted apical complexes. Note that the resolution is relatively high in the longitudinal direction (j, k), but in cross-section (i), elongation due to the missing wedge artifact is apparent (flattening results in orientation bias of the CFs). Labels and coloring as in Figure 4. Scale bars: 100 nm (in a-e and g); 10 nm (in i, j and k).

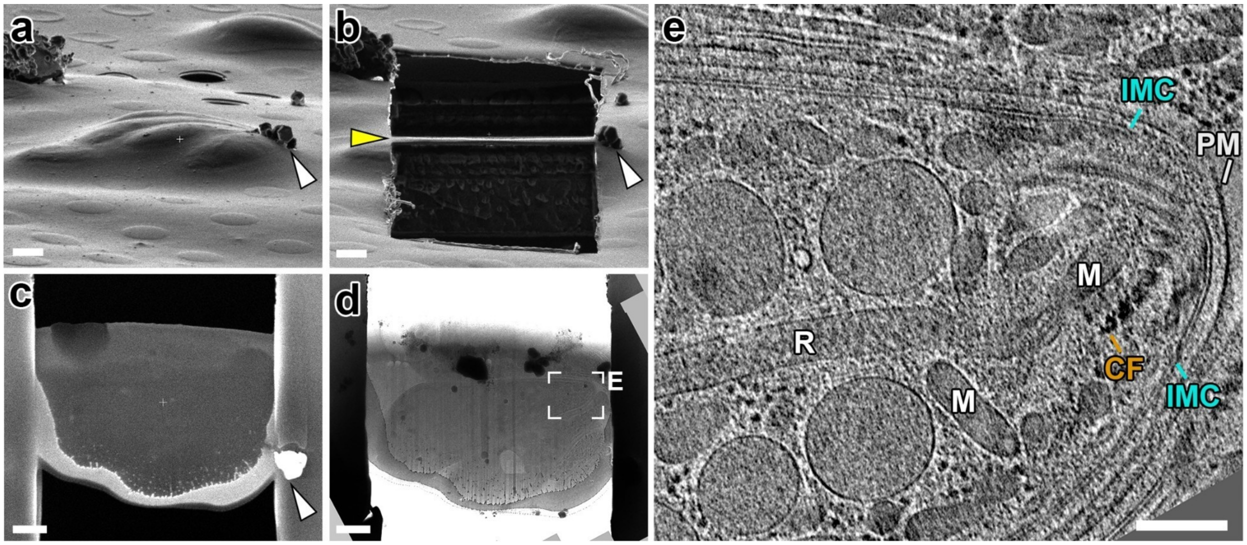

Supplementary Figure 3. The cryo-FIB milling workflow to generate lamella of plunge-frozen *Neospora caninum* cells for cryo-ET imaging. (a-c) Ion beam images (a, b) and a scanning-EM image (c) show the same grid area with targeted *N. caninum* cells before (a) and after (b, c) cryo-FIB milling in side (a, b) and top (c) views. Yellow arrowhead in (b) indicates the about 200 nm thick cryo-FIB lamella. For reference, the same contamination particle (white arrowheads) is indicated in all ion beam and electron beam images. **(d)** A cryo-TEM image of the same lamella shown in (c) with two clearly visible *N. caninum* cells. A tilt series was recorded of the apical region (white box) of the top parasite. **(e)** Representative tomographic slice of the reconstructed apical complex (boxed in (d)), showing the conoid in the retracted state. Labels: CF, conoid fiber; IMC, inner membrane complex; M, microneme; PM, plasma membrane; R, rhoptry. Scale Bar, 1 µm (in a-d); 200 nm (in e).

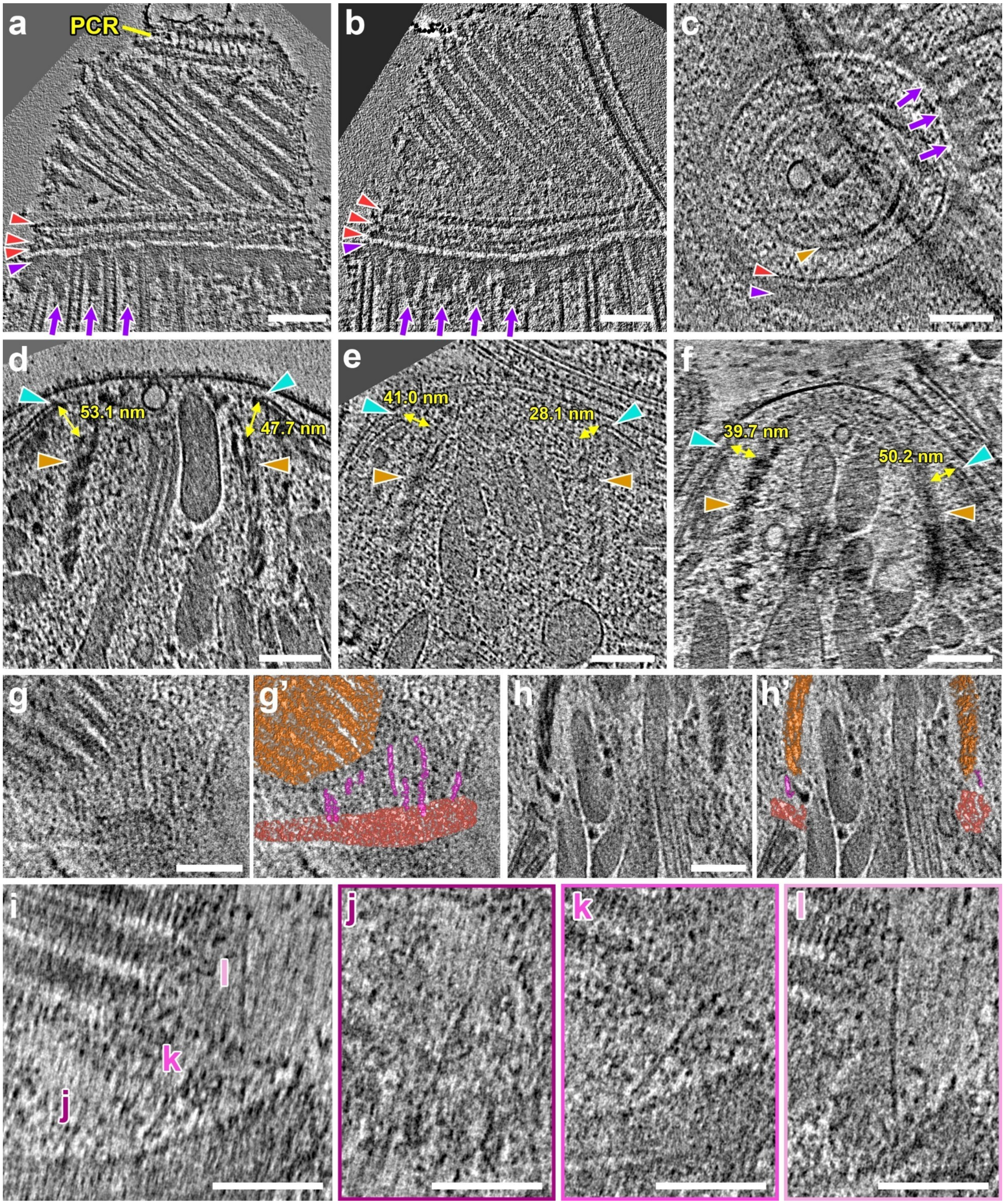

Supplementary Figure 4. Flexible interactions between the APRs and the conoid. (a, b) Tomographic slices highlight the APRs of detergent-extracted *Toxoplasma* tachyzoites. **(c)** Cross-sectional tomographic slice of the APR in the partially retracted conoid from a cryo-FIB-milled *Neospora* tachyzoite. Red arrowhead indicates one of the annular rings of the APRs, purple arrowhead and arrows indicate the AAD ring and AAD projections, respectively. **(d-f)** Representative tomographic slices through cryo-FIB milled conoid complexes in the retracted state. Measurements mark the closest distance between the IMC apical edge (cyan arrowheads) and the conoid fibers (orange arrowheads). **(g-l)** Tomographic slices (g,h: original: g',h': pseudo-colored) of protruded conoids show filamentous densities (magenta) with ~8 nm diameter and variable lengths that appear to

connect the conoid fibers (orange) to the APRs (red). Note that the orientation of these filaments slightly varies within the 3D reconstructions, therefore it is impossible to capture all connections in full length in a single tomographic 2D slice (see Figure 2g for example 3D rendering). In order to clearly visualize three exemplary filaments that are partially visible in (i; marked with j-l), multiple slices at slightly different z-depths and 3D orientations are shown in (j-l). Scale bars: 100 nm.

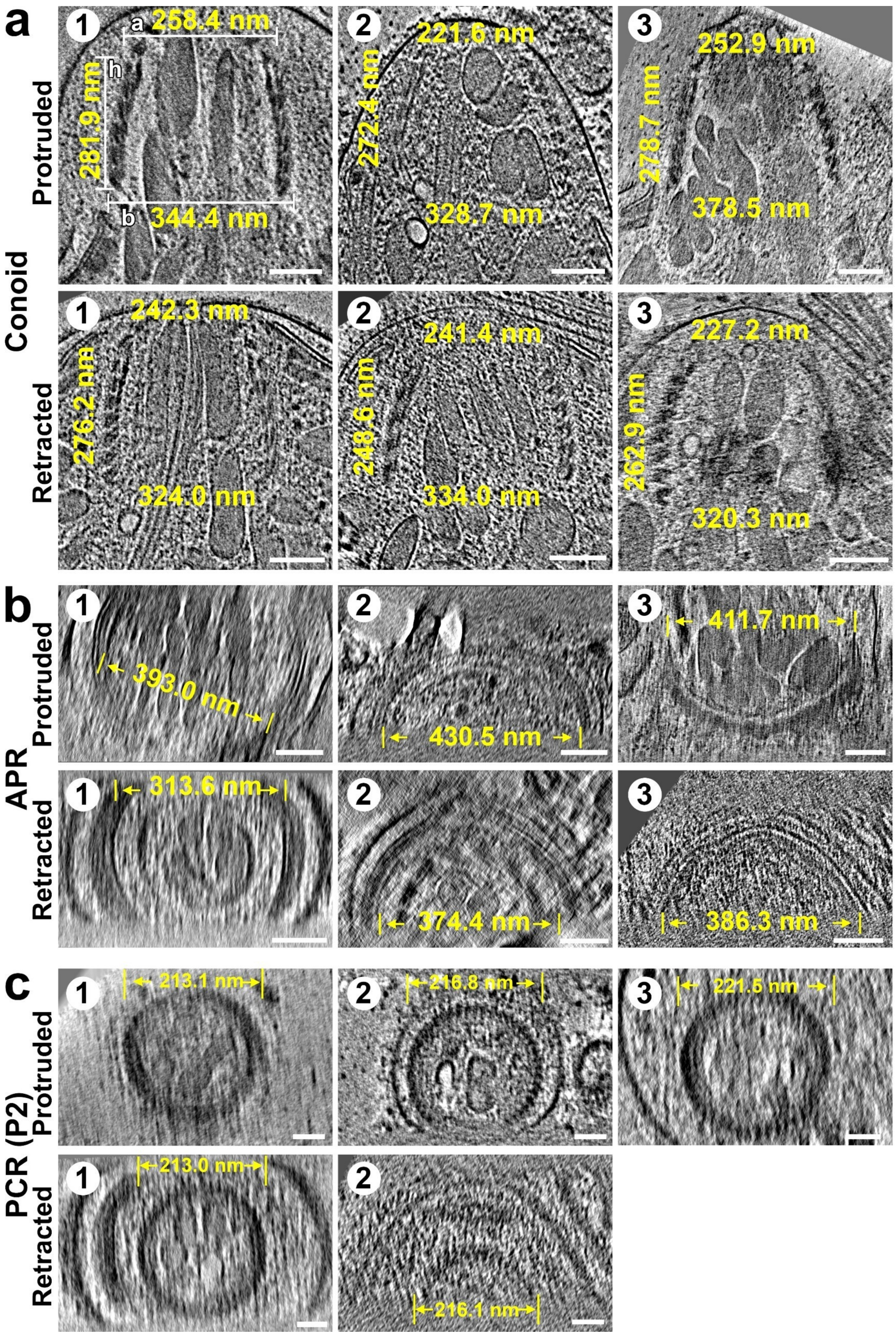

Supplementary Figure 5. Image gallery of the conoid complex, APRs and PCRs in the protruded and retracted states of *N. caninum*. (a) Tomographic slices of the cryo-FIB-milled apical complex in the protruded and retracted states. Measurements indicate the apical diameter [*a*], the basal diameter [*b*]) and the height [*h*] of the conoid structure (corresponds to analyses

shown in Figure 3e). **(b, c)** Cross-sectional tomographic slices of apical complexes show the diameters of the APRs (b) and the P2-PCR (c) in the protruded and retracted states. Scale bars: 100 nm (a and b); 50 nm (c).

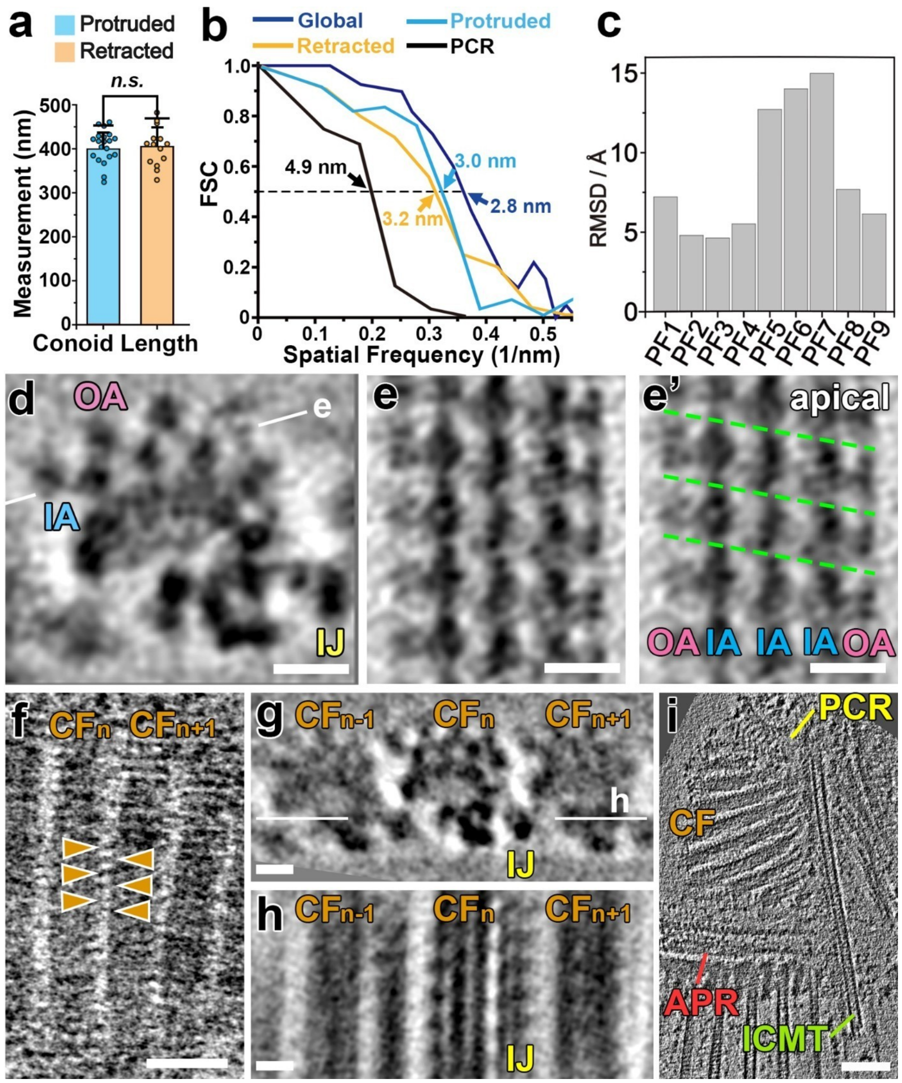

Supplementary Figure 6: Structural features of the subtomogram averages of the conoid fibers from cryo- FIB milled *Neospora* cells (a-h), and the ICMT of a detergent-extracted *Toxoplasma* tachyzoite (i). (a) The lengths of the conoid fibers (n=20 and n=14 measurements for the protruded and retracted states, respectively) are unchanged between the protruded and retracted states. Data were expressed as mean ± standard deviation. Statistical significance was calculated by two-tailed Student's t-test. n.s., not significant ($p>0.05$). **(b)** Using the Fourier shell correlation method, resolution estimates of the global*, protruded, and retracted average of the conoid fibers were measured at the PF4 region, whereas the resolution of the PCR average was estimated at the P2 ring (* combining tomograms from both protruded and retracted states). Dashed line and values indicate where the FSC curve intersects with the 0.5 criteria. **(c)** RMSD values comparing the relative position and orientation of each protofilament in the pseudo-atomic models after fitting tubulin dimers to the averaged conoid fiber in the retracted and protruded states (see corresponding Figure 4e). **(d-e')** Cross-sectional (d) and longitudinal (e: original, e': annotated) tomographic slices of the global subtomogram average of the conoid fibers show the pitch (indicated by green lines) along neighboring MAPs (e, e'). White lines in (d) indicate the positions of the longitudinal view in (e). **(f-h)** Tomographic slices through the conoid filaments in longitudinal orientation (f) show ~8-nm-spaced linkages (orange arrowheads) between adjacent conoid fibers (CF_{n-1}, CF_n and CF_{n+1}). In cross-sectional (g) and longitudinal (h) tomographic slices of the global subtomogram average of the conoid fibers (g, h;

depicted are three neighboring conoid fibers) these linkages can be seen as the inner junction (IJ) MAPs between PF3 of conoid fibers CF_n and PF9 of the neighboring CF_{n+1}. **(i)** Longitudinal tomographic slice of the apical complex of a detergent-extracted *Toxoplasma* tachyzoite shows the ICMT extending from the conoid apex passed the APRs. Scale bars: 10 nm (in d-e, g-h); 50 nm (in f); 100 nm (in i).

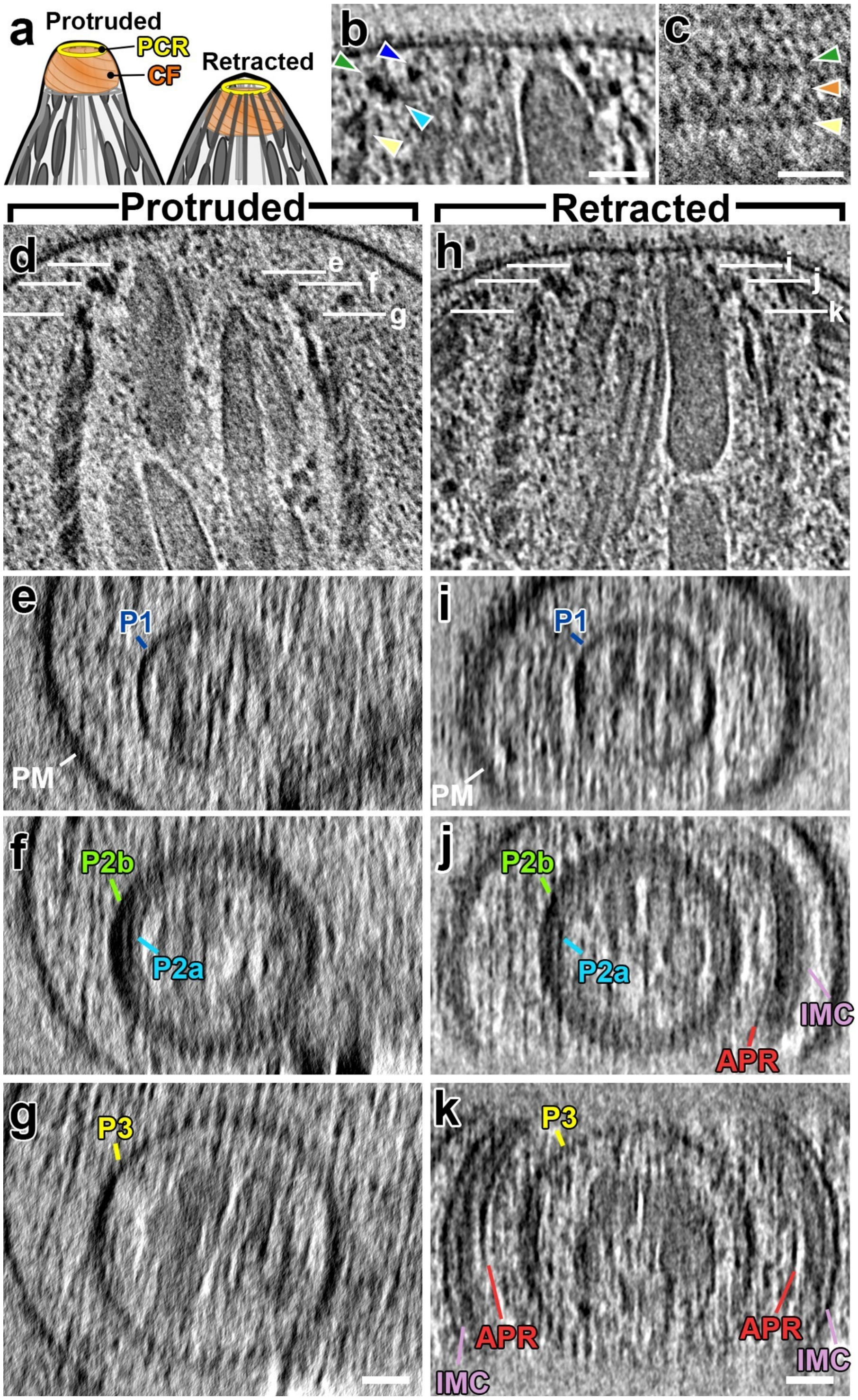

Supplementary Figure 7. The PCR complex consists of three layers of rings. (a) Cartoon highlighting the relative positions of the PCRs and CFs in the apical complex. **(b, c)** Tomographic slices through the top of the apical complex in longitudinal (b) and tangential (c) orientations reveal different components of the PCRs, including the apical P1 (highlighted by blue arrowheads), P2a (cyan), P2b (green), P3 (yellow) and the linker (orange) between P2b and P3. **(d-k)** Longitudinal (d, h) and cross-sectional tomographic slices (e-g, i-k) through the top of the cryo-FIB-milled *N. caninum* apical complex in either protruded (d-g) or retracted states (h-k) reveal that the PCR complex is composed of at least three distinguishable annular structures that we name from apical to basal: P1 (blue), P2 – with an interior P2a (cyan) and an exterior P2b

(green), and P3 (yellow). White lines in (d and h) indicate the positions of the corresponding cross-sectional views. Scale bars: 50 nm (in b, c, g, k, also valid for d-j).

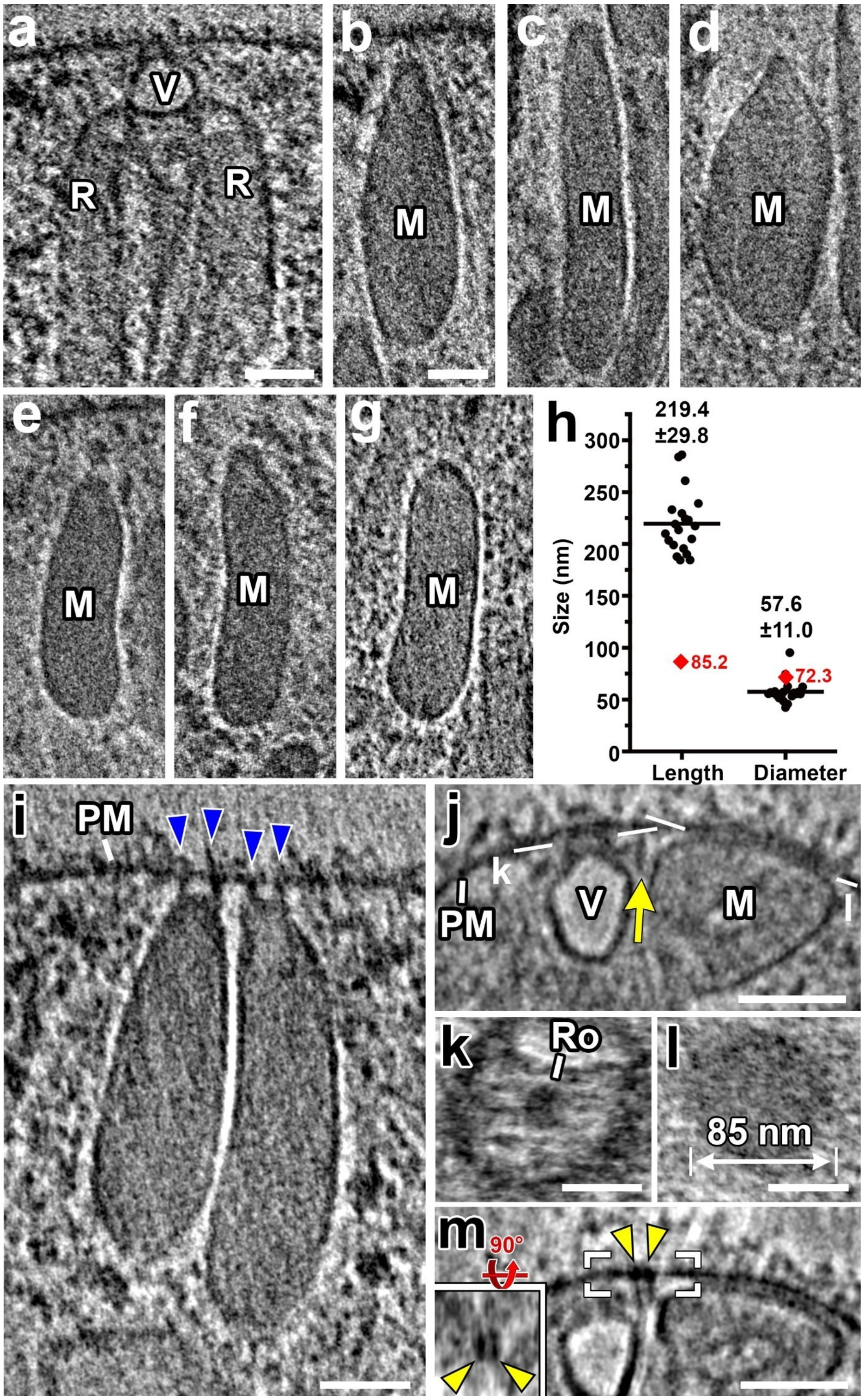

Supplementary Figure 8. Structural details about the secretory organelles within the apical complex show that micronemes have a regular shape and size, and dock to the plasma membrane at sites distinct from but organized with the rhoptry-associated vesicle. (a) Tomographic slice displaying a membrane-docked apical vesicle (V) that associates with the apical tips of 2 rhoptries (R). **(b-g)** Representative tomographic slices of micronemes in cryo-FIB-milled *Neospora* tachyzoites highlight the fairly consistent shapes and polar morphology of these organelles; apical is oriented towards the top. **(h)** Distribution of lengths and diameters of the micronemes from the cryo-ET reconstructions. Numbers are averages ± standard deviations (n=20 micronemes). Red diamonds are measurements from the PM-docked microneme in Figure 7j, k, and Supplementary Figure 8j-m. Note that the length of this microneme is significantly less

than the mean. **(i)** A tomographic slice showing two micronemes that connect to the plasma membrane (PM) through linkers (blue arrowheads), projecting from the corners of their flattened apical cap. **(j-m)** Tomographic slices provide side (j, m) and top views (k-l) of a microneme (M) and a rhoptry-associated vesicle (V) that are docked to the plasma membrane (PM) side-by-side (see also Figure 7j, k). The docking sites are distinct, i.e. the vesicle docks through the rosette (Ro in k) and the microneme via a contact site in a circular region of ~85 nm in diameter (l) to the plasma membrane. Despite the special separation, the location of docking appears to be organized in close proximity, likely through a long ridge (yellow arrow in (j)) that runs between the secretory organelles, and is tethered to a bi-partite anchor in the plasma membrane (yellow arrowheads in (m) and inset); the (m inset) shows the boxed area in (m) with the membrane anchor in top view. Scale bars: 50 nm (a, b, i-m; scale bar in b, also valid for c-g).

**Tomographic data collection**	**Cryo-FIB milled *Neospora caninum***	**Detergent-extracted *Toxoplasma gondii***
Microscope	Titan Krios G2i	Titan Krios G2i
Voltage (keV)	300	300
Nominal Magnification	26,000	26,000
C2 aperture	100	100
Objective aperture	Volta phase plate	Volta phase plate
Collection software	SerialEM	SerialEM
Tilt range	-56° to +56° (dose symmetric)	-56° to +56° (dose symmetric)
Tilt interval	Every 2°	Every 2°
Total no. of images per tilt series	57	57
Electron exposure per tilt ($e^-/Å^2$)	1.69	1.69
Total electron exposure per tilt series ($e^-/Å^2$)	96	96
Exposure rate (e^-/pixel/s)	28	28
Detector	Gatan K3 post Bioquantum energy filter	GatanK3 post Bioquantum energy filter
Calibrated pixel size (Å)	3.151	3.151
Defocus (µm)	-0.5	-0.5
Data acquisition software	SerialEM	SerialEM
**Reconstruction**	**Cryo-FIB milled *Neospora caninum***	**Detergent-extracted *Toxoplasma gondii***
Number of tomograms	20	11
Reconstruction software	IMOD	IMOD
Reconstruction method	Fiducial alignment and weighted back-projection	Fiducial alignment and weighted back-projection
Segmentation and visualization software	IMOD and Chimera	IMOD and Chimera

**Subtomogram averaging**	**Conoid fiber (*Neospora,* cryo-FIB milled)**	**Conoid fiber (*Toxoplasma,* detergent)**	**Pre-conoid ring (*Neospora,* cryo-FIB milled)**
Number of tomograms	4	2	4
Modeling programs in IMOD	addModPts	addModPts	spikeInit
Total number of extracted subtomograms	1935	692	187
Final averaged subtomograms	1881	692	180
Symmetry imposed	C1	C1	C1
Resolution (nm, FSC at 0.5)	2.8	3.1	4.9
Subtomogram averaging software	PEET	PEET	PEET

Supplementary Table 1: Tomographic data collection, reconstruction, and subtomogram averaging parameters.

# Tomograms	Description [sample, functional states…]	
**20**	**All tomograms of cryo-FIB milled *N. caninum***	
13/20	Protruded state, side-view milling	
7/20	Retracted state, side-view milling (3) and top-view milling (4)	
	**Structures**	**Representative figures**
20/20	IMC, PCR, APR, ICMT, micronemes, rhoptries, vesicles, apical sheet	Figures 2b, 2c, 5b-c, 5e-f, 7a-c, 7h, 7i; Sup. Figures 6f, 7b-c, Sup. Figure 8a-g
13/20	Protruded conoid, actin-like filaments	Figures 1c, 2d-g, 3a, 3c, 3f, 3j-k, 6a, 6f; Sup. Figures 4 g-l, 5a-c, 7d-g
7/20	Retracted conoid	Figures 1b, 1e, 3b, 3d, 3g, 3l-m, 6b, 6g; Sup. Figures 4c-f, 5a-c, 7h-k;
1/20	ICMT triple-MT	Figure 7f
1/20	Microneme connecting with PM	Sup. Figure 8i
1/20*	Microneme docking with PM	Figure 7j-k; Sup. Figure 8j-m
**11**	**All tomograms of detergent-treated *T. gondii*, protruded state**	Sup. Figures 2, 4a-b, 6i

*The case of microneme docking with PM was visualized in the state of protruded conoid

Supplementary Table 2: Summary of structures found in tomograms
