# Supplementary material for: Cryo-tomography reveals rigid-body motion and organization of apicomplexan invasion machinery
Source: Nat Commun. 2023 Mar 30;14:1775. doi: 10.1038/s41467-023-37327-w (PMC10063558; doi:10.1038/s41467-023-37327-w)
Supplement: Supplementary file 3 — Description of Additional Supplementary Files [file 41467_2023_37327_MOESM3_ESM.pdf]

Description of Additional Supplementary Files

File name: Supplementary Movie 1
Description: In situ cryo-ET reconstruction revealed the native structure of the N. caninum apical complex in the protruded state**.** Animated tomographic slices through a representative tomogram present and annotate first the cytoskeletal structures and secretory machinery in the apical complex (also shown in Figure 1d, 2d, 3a and 6a), followed by the 3D segmentation and visualization of the protruded invasion complex. Subsequently, the 3D isosurface rendering of the complete conoid (assembled from the subtomogram averaged conoid fibers) are shown in the side and bottom views, ending with a zoom-in view highlighting the Cshaped conoid fibers that open towards the inside of the conoid.
